# Investigation into Effective Factors Affecting the Self-Esteem of University Students Using a PLS-SEM Model

**DOI:** 10.3390/bs16101959

**Published:** 2026-10-21

**Authors:** Sengul Cangur, Fisun Kaşkır Kesin

**Affiliations:** 1 Department of Biostatistics and Medical Informatics, Faculty of Medicine, Düzce University, 81620 Düzce, Türkiye; 2 Division of Property Protection and Security, Vocational School of Social Sciences, Düzce University, 81620 Düzce, Türkiye

**Keywords:** self-esteem, food craving, social appearance anxiety, body mass index, partial least squares structural equation modelling

## Abstract

In this study, we investigated the associations among university students’ self-esteem, social appearance anxiety, food craving acceptance, willingness to consume a healthy diet, body mass index, subjective evaluation of weight change, and fast-food intake level. Data from 387 students were collected using an individual information form, the Food Craving Acceptance and Action Scale, the Social Appearance Anxiety Scale, and the Rosenberg Self-Esteem Scale, and analysed using bootstrapped partial least squares structural equation modelling (PLS-SEM). Self-esteem was negatively associated with social appearance anxiety and positively associated with willingness to consume a healthy diet (*p* < 0.05). Although the direct association between body mass index and self-esteem was non-significant (*p* > 0.05), significant negative indirect associations emerged, through willingness to consume a healthy diet and/or social appearance anxiety (*p* < 0.05). Contrary to expectations, a significant negative association was found between food craving acceptance and willingness to consume a healthy diet (*p* < 0.05). Self-esteem was indirectly associated with fast-food intake level and food craving acceptance negatively, and with subjective evaluation of weight change positively. Despite its high complexity and low explanatory power, the model achieved medium PLS predictive power, demonstrating some out-of-sample predictive relevance for the majority of indicators within the current PLSpredict framework. These findings are inherently exploratory, serving as a foundational framework for future longitudinal research while offering valuable insights for professionals providing health and psychological guidance to university students.

## 1. Introduction

Self-esteem reflects an individual’s sense of self-worth and respect (Mann et al., 2004). University years represent a critical developmental phase in which self-esteem is closely monitored and evaluated (Zafar et al., 2012), with academic achievement, talent display, body image, and social acceptance frequently examined as key correlates (Ozcan et al., 2013). While high self-esteem is conceptually linked to positive psychological traits such as optimism, self-confidence, achievement motivation, and resilience, low self-esteem is associated with patterns like insecurity, anxiety, fear of criticism, and social withdrawal (Mann et al., 2004). During developmental transition periods, individuals frequently show patterns of low self-esteem that co-occur with contexts characterized by academic failure, body image concerns, family pressure, economic hardships, and future uncertainty (Abdoli et al., 2025; Mann et al., 2004; Ozcan et al., 2013).

Food craving—a strong desire for a specific food—is experienced by over 90% of adults (Abdella et al., 2019; Hallam et al., 2016). When uncontrolled, multidimensional cravings are frequently associated with compromised health, appearance, and well-being (Taylor, 2019; Chao et al., 2019). Researchers examine these behaviours through two main frameworks: the traditional Elaborated Intrusion Theory (EIT) (Kavanagh et al., 2005) and third-wave approaches like Acceptance and Commitment Therapy (ACT) (Hayes et al., 2003). While EIT posits that elaborating on sensory images co-occurs with overeating, ACT suggests that suppressing urges expands their mental space; conversely, accepting them as transient somatic events is conceptually linked to the psychological flexibility needed for healthy eating (Taylor, 2019; Juarascio et al., 2011). Thus, food craving acceptance reflects one’s tolerance of distressing internal states, whereas craving action relates to the willingness to maintain healthy eating goals despite them (Taylor, 2019; Juarascio et al., 2011). Because university students living away from home frequently experience significant dietary shifts (El Ansari et al., 2012) and given the scarcity of research linking self-esteem and food craving acceptance and action (Manchón et al., 2021; Murray et al., 2022), investigating these associations in this population remains a critical research gap.

University years represent a critical period of social identity restructuring, during which physical appearance and the desire for social acceptance are prominent features (Kaskir Kesin, 2025). Students frequently attempt weight management during this period (Neumark-Sztainer et al., 2018). While the literature reports mixed findings regarding the relationships of body mass index, social appearance anxiety, and self-esteem with food cravings and eating behaviours (Abdella et al., 2019; Abdoli et al., 2025; Juarascio et al., 2011; Manchón et al., 2021; Hamurcu et al., 2023; Myers et al., 2018; Kaskir Kesin, 2025; Gobel et al., 2023; Chao et al., 2019; ALAhmari et al., 2019; Sanlier et al., 2018), these variables remain central to understanding student eating patterns. Social appearance anxiety—defined as the distress arising from the fear that one’s physical attributes, weight, and build will be negatively evaluated by others (Hart et al., 2008)—is frequently observed in relation to developmental vulnerabilities during young adulthood. Frequently associated with body dissatisfaction, eating disorders, and self-esteem (Mastro et al., 2016; Mancuso, 2016), social appearance anxiety is associated with psychological well-being. Consequently, examining the complex associations among social appearance anxiety, self-esteem, and food-related behaviours in university students remains a crucial endeavour in behavioural research (Kaskir Kesin, 2025; Gobel et al., 2023; Khan, 2024; Duran & Tras, 2022).

Smithson and Hill (2017) noted that findings regarding the relationships between food craving, weight control, and dieting success remain inconsistent. Given that subjective perceptions of recent weight fluctuations are associated with self-evaluations, and building on evidence that young adults frequently engage in weight management efforts during critical developmental stages (Neumark-Sztainer et al., 2018; Myers et al., 2018; Boswell & Kober, 2016), we hypothesised significant associations among subjective assessment of weight change, willingness to consume a healthy diet, and social appearance anxiety. Consequently, the interplay among recent weight changes, food craving, social appearance anxiety, and their indirect associations with self-esteem warrants thorough investigation among university students.

During university years, shifts in eating habits and easy access to fast-food co-occur with an increased intake of high-calorie foods (Basyah et al., 2025). Research indicates that young adults exhibit a higher propensity for fast-food, sweets, and high-fat cravings compared to older demographics (Abdella et al., 2019; Neumark-Sztainer et al., 2018), behaviours that are frequently associated with weight gain and related health risks (Smithson & Hill, 2017; Tang & Lee, 2024). Given that fast-food intake level is significantly associated with one’s willingness to consume a healthy diet, evaluating the indirect associations between weekly fast-food intake levels, social appearance anxiety, and self-esteem becomes a critical objective in understanding student eating behaviours.

Complex relationships exist among self-esteem, social appearance anxiety, and food craving dynamics. While some studies report significant relationships between food craving control and self-esteem (Manchón et al., 2021), others find no such associations (Sanlier et al., 2018). Furthermore, based on psychological flexibility, positive relationships were identified between negative body image and the acceptance of the desire for healthy eating and food cravings (Juarascio et al., 2011). Regarding statistical associations between social appearance anxiety and self-esteem, findings remain mixed, highlighting both direct and indirect (Kaskir Kesin, 2025; Gobel et al., 2023) and non-significant associations (Khan, 2024), alongside significant associations with emotional and restrictive eating behaviours (Duran & Tras, 2022).

Within this context, body mass index, subjective evaluation of weight change, and fast-food intake levels are posited to be indirectly associated with self-esteem. Going beyond simple theory confirmation to account for variance and predictive power, in this study, we investigate the direct and indirect associations among university students’ self-esteem, social appearance anxiety, food craving acceptance, willingness to consume a healthy diet, body mass index, subjective evaluation of weight change, and fast-food intake levels using bootstrapped partial least squares structural equation modelling (PLS-SEM).

## 2. Theorical Framework, Proposed Conceptual Model, and Hypothesis Development

**Figure 1:**
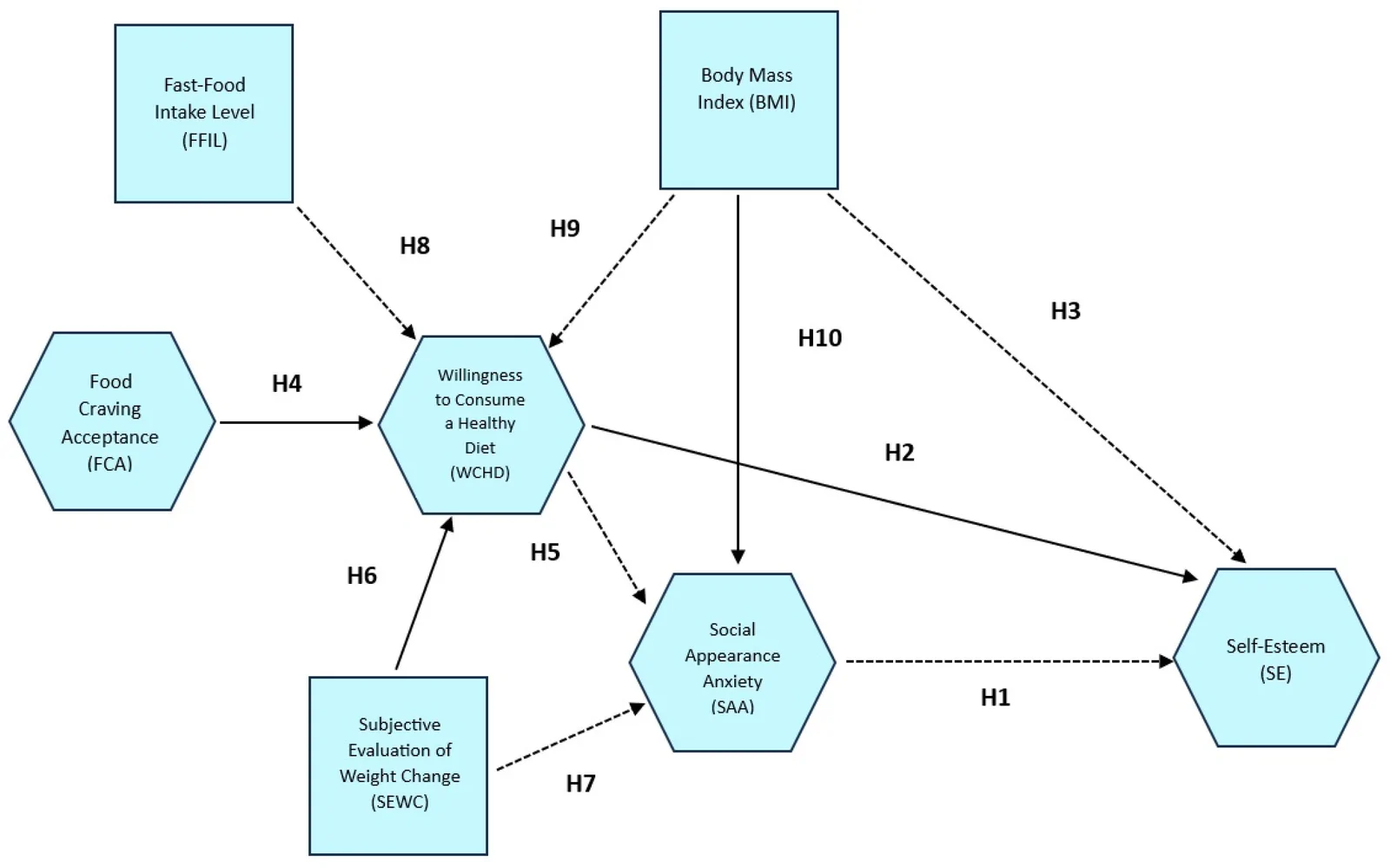
Proposed conceptual model (H: hypothesis; straight line arrow: positive relationship; straight dashed line arrow: negative relationship).

The proposed conceptual model and hypotheses were developed based on the underlying theories and theoretical models. The conceptual model of this study is shown in Figure 1.

### 2.1. Self-Esteem and Social Appearance Anxiety

The relationship between self-esteem and social appearance anxiety can be grounded in several foundational frameworks: self-presentation and impression management theory (Leary & Kowalski, 1990; Leary, 1996), self-discrepancy theory (Higgins, 1987), the need to belong (Baumeister & Leary, 1995), and sociometer theory (Leary & Baumeister, 2000). According to self-presentation and impression management theory, individuals reporting high social appearance anxiety frequently exhibit impression motivation and an intense fear of negative evaluation regarding their physical attributes, which correlate with lower self-esteem and self-worth. Similarly, self-discrepancy theory posits that when individuals experiencing social appearance anxiety perceive a wide gap between their physical reality and internalised societal standards (the ideal or ought self), this discrepancy is associated with chronic worthlessness and diminished self-esteem. Furthermore, from the perspectives of the need to belong and sociometer theories, the chronic fear of social rejection frequently accompanying social appearance anxiety may be interpreted within the framework of a compromised internal sociometer, thus co-occurring with lower levels of self-esteem. Grounded in these theoretical foundations, the conceptual model proposes the following hypothesis:

**H1.**  *Social appearance anxiety is negatively associated with self-esteem*.

### 2.2. Self-Esteem and Willingness to Consume a Healthy Diet

The association between self-esteem and willingness to consume a healthy diet is grounded in self-determination theory (Ryan & Deci, 2000) and contingent self-worth theory (Crocker & Park, 2004). According to self-determination theory, when autonomous motivation toward healthy eating—reflecting bodily self-respect and core personal values—co-occurs with the fulfillment of basic psychological needs (autonomy, competence, and relatedness), this pattern corresponds theoretically to stable, high self-esteem. Conversely, when eating behaviours are observed alongside controlled extrinsic pressures (e.g., social validation or guilt avoidance), this externalized motivational profile aligns with lower self-esteem. Similarly, contingent self-worth theory suggests that while tying one’s worth to rigid dietary adherence is empirically linked to unstable self-evaluations, genuine intrinsic commitment to healthy eating co-exists with higher self-efficacy and positive self-regard. Because autonomous healthy eating practices are conceptually mapped as patterns that generally correspond to psychological well-being in theoretical models, the conceptual model proposes the following hypothesis:

**H2.**  *Willingness to consume a healthy diet is positively associated with self-esteem*.

### 2.3. Self-Esteem and Body Mass Index

The association between body mass index (BMI) and self-esteem can be explained through the sociocultural theory and tripartite influence model (Thompson et al., 1999; Thompson & Stice, 2001), Harter’s model of self-worth (Harter, 1993), and the framework of weight stigma (Puhl & Brownell, 2006). According to sociocultural perspectives, specific physical standards—such as a slender or athletic physique—are idealised in modern societies, and deviations from these ideals often co-occur with negative social feedback and weight stigma. To the extent that individuals internalise these societal standards, a high BMI is perceived as a personal flaw, which is associated with lower self-esteem. Furthermore, from Harter’s (1993) developmental perspective, global self-worth corresponds to success across various social domains and the personal importance attached to them. Given that physical appearance serves as a dominant domain for self-evaluation, an elevated BMI co-occurs with body dissatisfaction and aligns with diminished global self-esteem when self-worth is contingent upon physical attractiveness (Cooley, 1902; Harter, 1993). In addition, weight stigma—defined in behavioural sciences as the phenomenon wherein individuals adopt societal weight-related prejudices and direct them toward themselves—is theoretically considered to run parallel to and statistically account for variance within this relationship (Puhl & Brownell, 2006; Vartanian & Smyth, 2013). Grounded in this theoretical framework, the following hypothesis is proposed:

**H3.**  *Body mass index is negatively associated with self-esteem*.

### 2.4. Food Craving Acceptance and Willingness to Consume a Healthy Diet

The relationship between food craving acceptance and the willingness to consume a healthy diet is examined within the framework of the EIT and cognitive flexibility paradigm (Kavanagh et al., 2005), as well as ACT and CBT paradigms (Hayes et al., 2003). The EIT paradigm posits that intense urges to eat are linked to involuntary sensory images and co-occur alongside overeating behaviours as individuals elaborate on these images. Within the ACT framework, attempts to suppress unwanted impulses, food cravings, or hedonic hunger signals are observed in a paradoxical pattern, which frequently co-occurs with an expansion of the mental space occupied by these thoughts. When individuals cease to perceive these impulses as threats and instead accept them as transient mental or somatic events, this psychological posture aligns with the psychological flexibility necessary to maintain a willingness to consume a healthy diet. Since the Food Craving Acceptance and Action Questionnaire (Juarascio et al., 2011) measures food-related psychological flexibility, and drawing upon ACT and cognitive–behavioural principles, the following hypothesis is proposed:

**H4.**  *Food craving acceptance is positively associated with the willingness to consume a healthy diet*.

### 2.5. Willingness to Consume a Healthy Diet and Social Appearance Anxiety

When the relationship between the willingness to consume a healthy diet and social appearance anxiety is grounded in self-determination theory and the concept of intrinsic motivation (Ryan & Deci, 2000) and the tripartite influence model (Thompson et al., 1999; Thompson & Stice, 2001), maintaining a healthy diet out of respect for one’s own body and an awareness of self-care—rather than to seek the approval of others—is theoretically linked to an intrinsic motivational pattern. This internal alignment is associated with lower social appearance anxiety in a manner that co-occurs with elevated psychological markers of resilience against criticism or negative evaluations from the outside world. In this context, hypothesis H5 of the conceptual model is proposed below:

**H5.**  *Willingness to consume a healthy diet is negatively associated with social appearance anxiety*.

### 2.6. Subjective Evaluation of Weight Change and Willingness to Consume a Healthy Diet

The association between the subjective evaluation of weight change and the willingness to consume a healthy diet is grounded in the theory of planned behaviour (Ajzen, 2005), learned helplessness theory (Seligman, 1975), and the restrained eating and restraint cycle models (Herman & Polivy, 1980). Positive perceptions of weight change co-occur alongside stronger intentions to eat healthily and correspond conceptually to higher perceived behavioural control within the framework of planned behaviour, whereas negative perceptions—such as perceived weight gain or unsuccessful dieting—are associated with learned helplessness, diminished willpower, and motivational withdrawal. Furthermore, within the restraint cycle framework, these perceived failures are associated with patterns of abandoning healthy intentions and co-occur with hedonic eating for emotional regulation. Grounded in this theoretical framework, hypothesis H6 is proposed as follows:

**H6.**  *The subjective evaluation of weight change is positively related to the willingness to consume a healthy diet*.

### 2.7. Subjective Evaluation of Weight Change and Social Appearance Anxiety

The association between the subjective evaluation of weight change and social appearance anxiety is grounded in self-presentation and impression management theory (Leary & Kowalski, 1990; Leary, 1996), weight stigma (Puhl & Brownell, 2006; Vartanian & Smyth, 2013), and sociometer theory (Leary & Baumeister, 2000). Negative evaluations of weight change are linked to threats to an individual’s impression management efforts, and co-occur with a heightened fear of negative evaluation and chronic social appearance anxiety. Conversely, positive evaluations align with higher sociometer reading while corresponding to conceptually lower levels of social appearance anxiety. Grounded in this theoretical framework, hypothesis H7 is proposed as follows:

**H7.**  *Subjective evaluation of weight change is negatively associated with social appearance anxiety*.

### 2.8. Fast-Food Intake Level and Willingness to Consume a Healthy Diet

The association between the fast-food intake level and the willingness to consume a healthy diet is grounded in the operant conditioning model (Skinner, 1938), the theory of planned behaviour (Ajzen, 2005), and ego depletion theory (Baumeister et al., 1999). A high fast-food intake level is theoretically linked to habituated reward-seeking patterns via operant conditioning and aligns with lower scores on the attitude component within the framework of planned behaviour. Furthermore, frequent consumption patterns co-occur with patterns of reduced cognitive resources and diminished self-regulation capacity, which correspond conceptually to lower intrinsic motivation to maintain a healthy diet. Grounded in this theoretical framework, hypothesis H8 is proposed as follows:

**H8.**  *Fast-food intake level is negatively associated with the willingness to consume a healthy diet*.

### 2.9. Body Mass Index and Willingness to Consume a Healthy Diet

The association between BMI and the willingness to consume a healthy diet is grounded in the self-compassion and positive body image model (Homan & Tylka, 2015), restrained eating and restraint cycle models (Herman & Polivy, 1980), learned helplessness theory (Seligman, 1975), and the foundations of weight stigma (Puhl & Brownell, 2006). A high BMI co-occurs with societal aesthetic norms and weight stigma, patterns that frequently map alongside restrained eating or learned helplessness. These external pressures are associated with conceptual patterns where the desire for healthy eating co-exists alongside a punitive, guilt-laden obligation rather than a flexible lifestyle change. As extrinsic and guilt-driven motivation aligns with lower intrinsic drive, perceptions of nutrition as an arena of failure—rather than a tool for health—are observed alongside lower willingness to consume a healthy diet. Grounded in this theoretical framework, hypothesis H9 is proposed as follows:

**H9.**  *Body mass index is negatively associated with the willingness to consume a healthy diet*.

### 2.10. Social Appearance Anxiety and Body Mass Index

The association between BMI and social appearance anxiety can be explained based on self-presentation and impression management theory (Leary & Kowalski, 1990; Leary, 1996) and the weight stigma model (Puhl & Brownell, 2006). According to self-presentation theory, individuals are motivated to create a desirable image in the eyes of others. However, elevated levels of BMI co-occur with heightened risks of negative social evaluation within an individual’s social environment and in specific settings—such as gyms, restaurants, and beaches. These interrelated patterns are associated with higher levels of social appearance anxiety. In this context, hypothesis H10, included in the conceptual model, is proposed below:

**H10.**  *Body mass index is positively associated with social appearance anxiety*.

In addition, the indirect associations associated with these variables were examined, as hypothesized below:

**H11a.**  *Willingness to consume a healthy diet is indirectly associated with self-esteem through social appearance anxiety*.

**H11b.**  *Food craving acceptance is indirectly associated with self-esteem through willingness to consume a healthy diet*.

**H11c.**  *Food craving acceptance is indirectly associated with self-esteem through the serial pathway of willingness to consume a healthy diet and social appearance anxiety*.

**H11d.**  *Food craving acceptance is indirectly associated with social appearance anxiety through willingness to consume a healthy diet.*

**H11e.**  *Fast-food intake level is indirectly associated with self-esteem through willingness to consume a healthy diet*.

**H11f.**  *Fast-food intake level is indirectly associated with self-esteem through the serial pathway of willingness to consume a healthy diet and social appearance anxiety*.

**H11g.**  *Fast-food intake level is indirectly associated with social appearance anxiety through willingness to consume a healthy diet*.

**H11h.**  *Subjective evaluation of weight change is indirectly associated with social appearance anxiety through willingness to consume a healthy diet*.

**H11i.**  *Subjective evaluation of weight change is indirectly associated with self-esteem through willingness to consume a healthy diet*.

**H11j.**  *Subjective evaluation of weight change is indirectly associated with self-esteem through the serial pathway of willingness to consume a healthy diet and social appearance anxiety*.

**H11k.**  *Body mass index is indirectly associated with social appearance anxiety through willingness to consume a healthy diet*.

**H11l.**  *Body mass index is indirectly associated with self-esteem through social appearance anxiety*.

**H11m.**  *Body mass index is indirectly associated with self-esteem through willingness to consume a healthy diet*.

**H11n.**  *Body mass index is indirectly associated with self-esteem through the serial pathway of willingness to consume a healthy diet and social appearance anxiety*.

PLS-SEM can be employed to evaluate the specified model but not to adjudicate between it and alternative structures that may fit comparably, so the proposed configuration is a theoretically defensible model rather than a uniquely supported one.

## 3. Materials and Methods

### 3.1. Participants

As a result of the PLS-SEM power analysis using the inverse square root method, the minimum sample size required to detect a minimum expected standardised path coefficient of 0.15 with 80% power and *p* = 0.05 was 275 (Ansani & Rinallo, 2025). The population of this research study consisted of nearly 30,000 students enrolled at a university in 2025. The university had a total of 25 faculties and vocational schools with students. To select the sample using proportionate stratified sampling, the population was stratified according to faculties/vocational schools. Considering the number of students in each faculty, 12 faculties/vocational schools were randomly selected, namely, Medicine, Pharmacy, Health Sciences, Arts and Sciences, Education, Engineering, Sports Sciences, Tourism, Forestry, Agriculture and Natural Sciences, Social Sciences, and Vocational Schools. The sample consisted of 387 students recruited from these randomly sampled faculties and vocational schools who agreed to participate in the study between March and October 2025 and fully completed the form (note that students were not paid any fees).

### 3.2. Measures

All data were collected using an individual information form, the Rosenberg Self-Esteem Scale, the Food Craving Acceptance and Action Questionnaire, and the Social Appearance Anxiety Scale. Some of the data was obtained online, while the rest was collected through face-to-face interviews where students were asked to complete the form themselves between March and October 2025.

*Individual Information Form*: This form contained information on the students’ socio-demographic characteristics (age, gender, place of stay, income status, and education levels of mother and father) and some additional questions including status of sport participation, alcohol use status, smoking status, weight, height, healthy nutrition status, nutritional preference, subjective evaluation of weight change in recent years, fast-food intake level (week), and probiotic/prebiotic food intake level (week).

*Rosenberg Self-Esteem Scale*: In 1965, Rosenberg developed this scale to assess global self-esteem, which contains both positive and negative emotions. This scale is one-dimensional with 10 items (SE, items: SE1–SE10). The total score was calculated using a 4-point Guttman scale (from 1: strongly disagree to 4: strongly agree), one of various scoring methods. The reverse-coded items on the scale are items 2, 5, 6, 8, and 9. Higher scores show higher self-esteem. Cuhadaroglu (1986) assessed the Turkish validity–reliability of this scale using 0–3 point scoring, and Cronbach’ alpha was 0.88.

*Food Craving Acceptance and Action Questionnaire*: Developed by Juarascio et al. (2011), this scale assesses psychological flexibility regarding eating by measuring craving acceptance and the willingness to engage in healthy behaviors despite distress. The scale is a 6-point Likert-type scale (very rarely true: 1, rarely true: 2, sometimes true: 3, frequently true: 4: almost always true: 5, always true: 6) with 10 items, with a two-factor structure of “Willingness” and “Acceptance.” Items 4, 6, 7, and 9 of the scale are reverse-scored. The “Willingness” subscale assesses the individual’s desire to direct themselves to a healthy diet despite food-related feelings, thoughts, and cravings (items 1, 2, 3, 5, 8, and 10), while the “Acceptance” subscale assesses the individual’s acceptance of feelings, thoughts, and cravings related to food without attempting to control, change, suppress, or avoid intense food cravings, thoughts, and feelings (items 4, 6, 7, and 9) (Manchón et al., 2021). The Turkish validation study of this scale was performed by Hamurcu et al. (2023). A higher willingness score reflects individuals’ motivation for healthy eating, while higher acceptance score reflects the acceptance of intense food cravings without controlling/changing negative thoughts and feelings of individuals. Cronbach’s alpha coefficients for the total scale and willingness and acceptance subscales were found to be 0.92, 0.89, and 0.86, respectively (Hamurcu et al., 2023). We included food craving acceptance (FCA) and willingness to consume a healthy diet (WCHD) in this research model and represented the items of FCA and WCHD as FCA1-FCA4 and WCHD1-WCHD6, respectively.

*Social Appearance Anxiety Scale*: Hart et al. (2008) developed this scale to assess the emotional, behavioural, and cognitive anxieties experienced by an individual concerning their appearance. This scale has 16 items (SAA, items: SAA1-SAA16) with a 5-point Likert-type scale (from 1: not at all appropriate: 1 to 5: completely appropriate). Only item 1 is reverse-scored in this scale. The Turkish validity–reliability of the scale was assessed by Dogan (2010). Higher scores indicate higher social appearance anxiety. Cronbach’ alpha was 0.93 (Dogan, 2010).

Body mass index (BMI, kg/m^2^) was calculated from weight and height measures declared by the students. Subjective evaluation of weight change (SEWC) was categorized as 1 for positive effects and 0 for negative effects based on recent weight history. The weekly fast-food intake level (FFIL) was coded on a 4-point ordinal scale (1: never; 2: 1–3 times; 3: 4–6 times; 4: 7+ times), with pseudo-metric values (0, 2, 5, and 8) applied for sensitivity analysis.

### 3.3. Statistical Analysis

Mardia’s Kurtosis test, scatter plots, and the variance inflation factor (VIF < 5) approach (inner and outer models) were used to check the assumptions of multivariate normality, linearity of variables, and collinearity, respectively. In this study, we aim not only to confirm an existing theory (the proposed conceptual model) but also to explain the variance in the dependent variable and demonstrate predictive power. Furthermore, since the proposed conceptual model presented in Figure 1 is being tested for the first time in this specific combination and context, the study is partly exploratory and theory-developing/-extending in nature. The model possesses a complex structure due to the inclusion of numerous independent variables. Under these conditions, the PLS-SEM approach is superior to other SEM approaches (such as covariance-based SEM/CB-SEM) (Hair et al., 2021). Therefore, the research model was constructed using bootstrapped partial least squares structural equation modelling (PLS-SEM) with 10,000 iterations. The bootstrap standard errors, t-values, and 95% confidence intervals for this model were reported. Regarding indicator reliability, standardised factor loadings below 0.40 were directly removed from the model, whereas the model’s validity and reliability were jointly evaluated to decide whether to remove factor loadings between 0.40 and 0.70. Average variance extracted (good: AVE ≥ 0.50; acceptable: AVE < 0.50 if CRs ≥ 0.60) values were examined to check the convergent validity in the PLS-SEM measurement model. In addition, Cronbach’s alpha (good: CA ≥ 0.70; acceptable: 0.60 ≤ CA < 0.70) and composite reliability (good: CR ≥ 0.70; acceptable: 0.60 ≤ CR < 0.70) were reported for the internal consistency reliability of this model. The heterotrait–monotrait ratio with bootstrap 95% confidence interval (HTMT < 0.85) and Fornell–Larcker criterion (the square roots of AVEs > the correlations between the constructs) were assessed for discriminant validity. The effect sizes (small: 0.02 ≤ f2 < 0.15; medium: 0.15 ≤ f2 < 0.35; substantial: f2 ≥ 0.35) and R^2^ values (substantial: R^2^ ≥ 0.75; moderate: 0.50 ≤ R^2^ < 0.75; weak: 0.25 ≤ R^2^ < 0.50; very weak: R^2^ < 0.25) were used for the explanatory power of the structural model (Hair et al., 2021; Yilmaz & Surmelioglu, 2025). PLSpredict was used for the out-of-sample predictive assessment of the structural model (k-fold = 10). The root mean square error (RMSE) and mean absolute error (MAE) were recorded for PLS and Linear Model (LM) out-of-samples (high: the RMSEs and MAEs of PLS out-of-sample < the RMSEs of LM out-of-sample; medium: many RMSEs and MAEs of PLS out-of-sample < the RMSEs of LM out-of-sample; low: many RMSEs and MAEs of PLS out-of-sample > the RMSEs and MAEs of LM out-of-sample) (Shmueli et al., 2019; Hair et al., 2021). For the construct-level metric, the MSE and MAE values of in-sample (training) and out-of-sample (testing) were calculated to determine overfitting levels. Additionally, the standardised root mean squared residual (reasonable fit: SRMR < 0.08) was calculated to assess model fit. To test model robustness, a sensitivity analysis was performed using alternative pseudo-metric values for FFIL. All statistical analyses were performed using the RStudio 2026.09.0+147 programme. *p* < 0.05 was considered statistically significant.

## 4. Results

### 4.1. Some Characteristics of Participants

Of the 387 university students, 54.5% were female and 45.5% were male, with an average age of 20.59 ± 2.06. In total, 64.3% of students stated that they live in halls of residence, 61.5% said that their mothers are primary school graduates, 25.3% participate in sports, 38.2% eat healthily, and 89.7% do not consume alcohol. In total, 71.8% stated that they consume fast-food 1–3 times a week, and 59.9% stated that they consume probiotic/prebiotic foods 1–3 times a week. Some characteristics of the participants are given in detail in Table 1.

### 4.2. Evaluation of the Measurement and Structural Models

The distribution of data does not meet the multivariate normality (Mardia’s Kurtosis statistics = 45.09, *p* < 0.001). The estimates of model were calculated by performing the bootstrapped PLS-SEM approach. The initial model included four latent variables (SE, SAA, FCA, WCHD) with 36 items and three single-item observed variables (BMI, SEWC, FFIL), and the results of the model are given in Tables S1–S3 and Figure S1.

The standardised factor loadings of each item varied from 0.247 to 1.00 in the initial PLS-SEM model (Figure S1). The inner VIFs ranged from 1.03 to 1.18, indicating the absence of structural collinearity. Although certain outer VIFs slightly exceeded the threshold of 5 (SAA12 = 5.20; SAA13 = 5.35; SAA16 = 5.61), these indicators were retained to preserve the content validity of the reflective measurement model, as high indicator collinearity does not compromise structural path estimates. The findings of internal consistency reliability and convergent validity of this initial model are given in Tables S2 and S3. The Cronbach’s alpha (CA) and composite reliability (CR) of each factor were more than 0.71 and 0.73, respectively. The average variance extracted (AVE) values—that is, the convergent validity values—for each construct in the initial model ranged from 0.443 to 0.645 (Table S1). In addition, HTMT (95% CI) and the Fornell–Larcker values for discriminant validity are given in Table S2. The HTMT values varied between 0.014 and 0.589, and they were less than 0.85. The Fornell–Larcker values, which represent the squares root of AVEs, ranged from 0.666 to 0.803, and were larger than the correlations between the constructs. In addition, the direct, indirect, and total effects of the initial bootstrapped PLS-SEM model and hypothesis testing results are presented in Table S3.

After removing the FCA1 item (β = 0.247; *p* = 0.247; 95%CI: −0.240–0.494) of the food craving acceptance factor with a factor loading below 0.40, which was not significant, the reliability and validity values of the PLS-SEM model were reviewed. Moreover, when the impact of this removal on the content validity of the FCA construct was evaluated, the remaining indicators comprehensively represented all fundamental aspects and boundaries of the construct. The final PLS-SEM model included four latent variables with 35 items and three single-item observed variables. The findings of the internal consistency reliability and convergent validity of this model are given in Table 2. Cronbach’s alpha and composite reliability of each factor were more than 0.71 and 0.80, respectively. The AVEs representing the convergent validity of each construct in the model ranged from 0.518 to 0.645.

For discriminant validity, the Fornell–Larcker values and the HTMT with 95% CI are given in Table 3. The Fornell–Larcker values varied between 0.720 and 0.803 and were greater than the correlations between the constructs. The HTMT values varied between 0.014 and 0.589 and were less than 0.85. The inner VIFs were obtained to check the collinearity problem between factors in the PLS-SEM structural model. They were computed from the regression of SAA, WCHD, and BMI with SE as 1.177, 1.153, and 1.062, respectively; from the regression of WCHD, BMI, and SEWC with SAA as 1.068, 1.080, and 1.103, respectively; and from the regression of BMI, FCA, SEWC, and FFIL with WCHD as 1.064, 1.026, 1.075, and 1.033, respectively. These values are less than 5.

In evaluating the explanatory power of this structural model, the effect size (f2) of each latent variable on SE substantial for SAA (f2 = 0.347) and small for WCHD (f2 = 0.040); the effect sizes of WCHD, BMI, and SEWC on SAA were small (f2 = 0.094, f2 = 0.017, and f2 = 0.049, respectively); and the effect sizes of FCA, SEWC, FFIL, and BMI on WCHD were also small (f2 = 0.112, f2 = 0.039, f2 = 0.089, and f2 = 0.019, respectively). The R^2^ values for each endogenous construct were calculated as 0.354 for SE, 0.245 for WCHD, and 0.191 for SAA. The predictive power values of the final PLS structural model are given in Table 4. With few exceptions (SE1, SE5, and SE10), PLS yielded lower out-of-sample RMSEs and MAEs than LM. The construct-level overfitting values for SE, SAA, and WCHD were 0.019, 0.011, and 0.028, respectively (Table 4), and the SRMR model fit was 0.069 (SRMR < 0.08).

**Figure 2:**
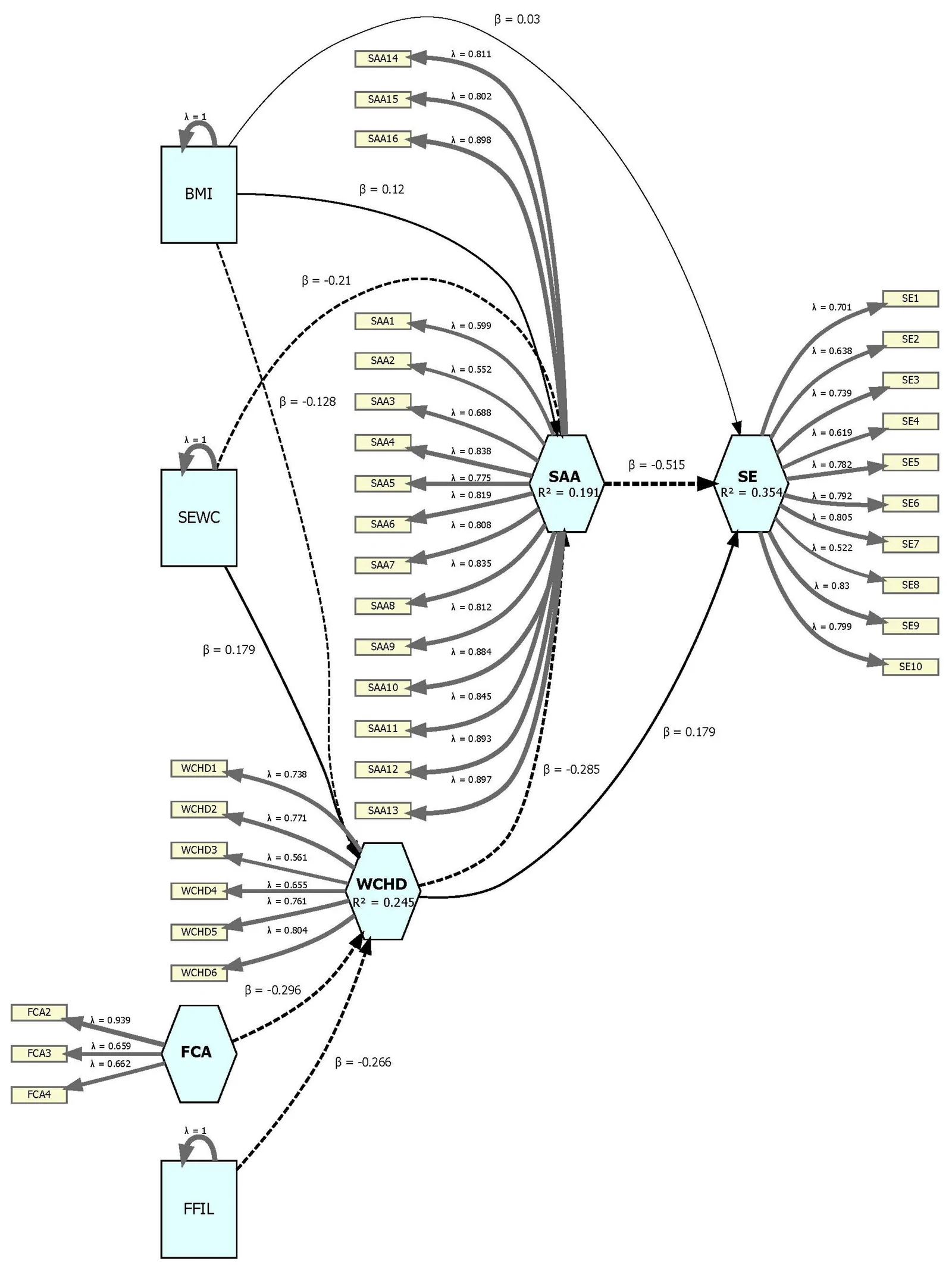
The final PLS structural equation model. λ: factor loading; β: standardised path coefficient; straight line arrow: positive relationship; straight dashed line arrow: negative relationship; SE: self-esteem; SAA: social appearance anxiety; FCA: food craving acceptance; WCHD: willingness to consume a healthy diet; BMI: body mass index; SEWC: subjective evaluation of weight change; FFIL: fast-food intake level.

Following the removal of the FCA1 item, the final PLS-SEM model is illustrated in Figure 2, while the direct, indirect, and total effects—along with the hypothesis testing results—are presented in Table 5. All item factor loadings were significant and ranged from 0.52 to 1.00 in the final PLS-SEM model (Figure 2). With the exception of H3 and H4, all proposed direct and indirect hypotheses were supported. Although a *p* < 0.001 was obtained for hypothesis H4, it was not accepted because the association within it had been predicted to be positive. Contrary to the formulated hypothesis, a significant negative association was found between food craving acceptance and willingness to consume a healthy diet (Table 5).

In this study, we found that self-esteem is positively related to the willingness to consume a healthy diet (β = 0.179; *p* < 0.001), while it is significant and negatively associated with social appearance anxiety (β = −0.515; *p* < 0.001). This coefficient indicates that a one-standard-deviation increase observed in variable social appearance anxiety is associated with a 0.515-standard-deviation decrease in self-esteem. These findings are consistent with the model. However, body mass index is not associated with self-esteem (β = 0.030; *p* = 0.449), which is inconsistent with the model. Moreover, significant negative associations were found between the willingness to consume a healthy diet and social appearance anxiety (β = −0.285; *p* < 0.001), fast-food intake level (β = −0.266; *p* < 0.001), and body mass index (β = −0.128; *p* = 0.004), whereas the willingness to consume a healthy diet was found to be positively correlated with the subjective evaluation of weight change (β = 0.179; *p* < 0.001). A positive relationship was observed between social appearance anxiety and body mass index (β = 0.120; *p* = 0.022), while a significant negative relationship was found between social appearance anxiety and the subjective evaluation of weight change (β = −0.210; *p* < 0.001). These results support our hypotheses and are consistent with the model (Table 5).

The evaluation of the total and indirect effects in the final bootstrapped PLS-SEM model revealed that all other total effects were statistically significant (*p* < 0.05; Table 5), with the exception of the total path coefficient from BMI to SE (*p* > 0.05). Specifically, the total path coefficient from WCHD to SE was 0.325 (*p* < 0.001), and the indirect path coefficient was 0.147 associated through SAA (*p* < 0.001). In other words, a one-standard-deviation difference in the willingness to consume a healthy diet score is associated with a direct difference of 0.325 standard deviations in self-esteem scores, while also reflecting an indirect association of 0.147 standard deviations along the hypothesised structural path. Furthermore, all indirect path coefficients from BMI to SE (−0.063 through SAA, −0.023 through WCHD, and −0.019 through WCHD and SAA) were significant and negative (*p* < 0.05), indicating lower levels of SE associated with higher levels of BMI through the other factors. All indirect path coefficients from SEWC to SE through WCHD and/or SAA were positive and significant (*p* < 0.001), whereas all indirect paths from FFIL and FCA to SE through WCHD and/or SAA were negative and significant (*p* ≤ 0.001; Table 5).

The results of the sensitivity analysis obtained using an alternative coding scheme (0, 2, 5, and 8) for FFIL were found to be consistent with the final PLS-SEM model.

## 5. Discussion

The associations among self-esteem, social appearance anxiety, food craving acceptance, willingness to consume a healthy diet, body mass index, subjective evaluation of weight change, and fast-food intake levels of university students were concurrently investigated with the PLS-SEM model in this research. We found that the final PLS measurement model demonstrated a good level of convergent validity (AVEs ≥ 0.50) and met the criteria for discriminant validity (HTMT < 0.85; FLc(s) > correlations between constructs). In addition, the construct reliability of the PLS measurement model (CAs ≥ 0.70; CRs ≥ 0.70) was at a good level, but the CA = 0.962 and CR = 0.966 values for social appearance anxiety slightly exceeded the critical threshold of 0.95, suggesting potential item redundancy. The structural model demonstrated reasonable fit according to the model fit index (SRMR = 0.069 < 0.08). The out-of-sample predictive assessment via PLSpredict indicated that the model achieved PLS predictive power, demonstrating some out-of-sample predictive relevance within the current PLSpredict framework. The findings provide evidence of predictive utility within this sample but should not be interpreted as evidence of broader population-level generalizability. While the model predicted self-esteem with higher accuracy relative to other constructs, it exhibited slightly lower predictive performance for social appearance anxiety, as social appearance anxiety was associated with other exogenous factors in a more complex manner. However, the path effect sizes were generally small with the exception of the self-esteem construct (f2 = 0.35), and the R^2^ values were generally at a weak level (0.19 to 0.35). The low values obtained for some measures may stem not only from psychological, environmental, or genetic factors excluded from the model but also from the model’s inclusion of three distinct single-item observed variables (BMI, SEWC, and FFIL) and the numerous complex relationships among constructs. Based on the literature (Hair et al., 2021; Diamantopoulos et al., 2012), it can be stated that the inability to isolate measurement error in single-item observed variables—and the resulting attenuation effect—may have suppressed the path coefficients and the R^2^ value.

The results of the sensitivity analysis conducted for fast-food intake levels were consistent with the final PLS-SEM model, confirming that the findings were not dependent on a specific coding choice.

We found that social appearance anxiety of university students is significantly and negatively associated with their self-esteem, and this association exhibited the greatest path coefficient in the model. An individual with high social appearance anxiety frequently reports a constant sense of threat regarding being perceived as inadequate or flawed by others; this theoretical pattern may be interpreted within theoretical frameworks concerning social evaluation, and is associated with avoidance behaviours and patterns of seeking approval in relationships, while also aligning conceptually with lower levels of self-esteem among individuals navigating potential negative judgments from others (Leary & Kowalski, 1990; Leary, 1996; Baumeister & Leary, 1995; Leary & Baumeister, 2000). This finding is parallel to the results of several researchers (Kaskir Kesin, 2025; Behera & Pandey, 2025; Liao et al., 2023; Colak et al., 2023; Lau & Idang, 2022; Hawi & Samaha, 2017; Topuz et al., 2019). Abdoli et al. (2025) demonstrated that university students with higher self-esteem exhibit less appearance dissatisfaction, a lower fear of weight gain, and diminished bodily detachment. Moreover, Gobel et al. (2023) and ALAhmari et al. (2019) showed that social appearance anxiety or perception of body dissatisfaction of female participants is negatively associated with their self-esteem. Nevertheless, Khan (2024) obtained no significant relationship between social appearance anxiety and self-esteem. This difference in results may stem from social norm differences as well as the use of a non-probability sampling approach. Other studies involving university students have also highlighted the crucial role of social norms and cultural differences in shaping these relationships (Abdoli et al., 2025). Furthermore, Gobel et al. (2023) showed that the indirect association of social appearance anxiety with self-esteem via BMI was positive–significant.

This study demonstrated that the self-esteem level of university students is significantly related to their willingness to consume a healthy diet. When individuals’ desire for healthy eating co-occurs with autonomous motivation, this motivational pattern maps alongside the fulfillment of basic psychological needs, which is theoretically aligned with higher self-esteem. Furthermore, an individual’s self-esteem is associated with a genuine internal commitment to healthy eating, rather than experiencing unstable self-evaluations associated with rigid dietary rules (Ryan & Deci, 2000; Crocker & Park, 2004). However, since this relationship can be bidirectional, higher levels of self-esteem are empirically observed alongside elevated levels of willingness to consume a healthy diet. This result is similar to that of Manchón et al. (2021). However, Juarascio et al. (2011) found no significant relationship between willingness to consume a healthy diet and self-esteem of individuals. The reason for the discrepancy between this result and ours may stem from methodological and contextual variations, such as differences in sample characteristics, measurement tools, or cultural contexts that shape dietary motivation. Furthermore, while the desire for healthy eating reflects a transient motivational state, self-esteem represents a general self-evaluation. Consequently, individuals may possess high self-esteem—shaped by environmental, biological, and sociocultural factors—even in the absence of a desire to maintain healthy eating habits. Conversely, an individual might fail to sustain their dietary intentions due to the psychological discomfort associated with cognitive dissonance. Moreover, as a result of cognitive dissociation (Ong et al., 2017), individuals with high health consciousness and moderate self-esteem may still harbour strong dietary intentions. In such instances, a statistically significant relationship between the willingness to consume a healthy diet and self-esteem may not be observed. Consequently, these explanations should be regarded as possible interpretations requiring future empirical investigation.

Even though the direct path between BMI and students’ self-esteem was non-significant in this study, the indirect paths from BMI to self-esteem were found to be significant through social appearance anxiety, through willingness to consume a healthy diet, and through a serial combination of both. This result indicates that, in human psychology, self-esteem is associated with much different dynamics independent of weight. Specifically, higher levels of BMI were found to be associated with lower self-esteem through social appearance anxiety. In societies where thinness is accepted as the ideal body standard, the finding that individuals with a high BMI experience elevated levels of state social appearance anxiety—and that this correlates negatively with their perception of self-esteem—is consistent with the literature (Kaskir Kesin, 2025); however, it should be noted that the direction of these relationships could be reciprocal (i.e., low self-esteem may also co-occur with higher levels of anxiety). A similar result was obtained through willingness to consume a healthy diet. Furthermore, higher BMI values were associated with lower self-esteem through the serial pathway involving willingness to consume a healthy diet and social appearance anxiety. In general, these indirect associations reflect patterns consistent with the structural model. Although our cross-sectional study does not definitively prove the causal relationship, the risk of negative social evaluation within an individual’s social circles co-occurs with elevated levels of BMI; exposure to prejudice and weight stigma is associated with social appearance anxiety (Titchener & Wong, 2015), which relates to lower self-esteem as it co-occurs with the avoidance of social settings (Leary & Baumeister, 2000). Similarly, the interpretation that an individual with a high BMI has low self-esteem—associated through willingness to consume a healthy diet—can also be grounded in psychological and behavioural theoretical constructs (such as self-compassion and positive body image, restrained eating and restraint cycle models, learned helplessness theory, and weight stigma). It is consistent with the literature that, in a society dominated by ideal body measures, individuals with a high BMI exhibit high levels of state anxiety and/or employ maladaptive coping strategies that relate to their eating habits—factors that show a negative connection with their perception of self-esteem (Kaskir Kesin, 2025; Tylka et al., 2014). However, an interaction between the variables may also be present. The results of this study are analogous to those of Kaskir Kesin (2025) for the direct and indirect associations of BMI on the students’ self-esteem through social appearance anxiety and Sanlier et al. (2018) for the non-significant association of BMI on the students’ self-esteem. However, ALAhmari et al. (2019) obtained a significant negative association between the BMI and self-esteem of female participants. They may have identified this significant relationship because female participants placed more importance on their weight and BMI. Moreover, Gobel et al. (2023) stated that BMI had a near-zero positive–significant association with self-esteem.

In this study, the hypothesis positing a positive–significant association between food craving acceptance and the willingness to consume a healthy diet was counterintuitive to our theoretical expectations, revealing a negative association (*p* < 0.001). We anticipated a positive association based on theoretical assumptions—such as the traditional EIT (Elaborated Intrusion Theory) paradigm, where intense eating urges co-occur with involuntary sensory images. As individuals dwell mentally on these images, the urges are associated with overeating behaviours. However, the findings indicate the contrary. Rather than interpreting this unexpected negative association as a global deficit in sample-wide food-related psychological flexibility, it highlights how acceptance-based processes may manifest differently within specific behavioural contexts. Additionally, given that 61.8% of the students possessed unhealthy eating habits, this statistical pattern may explain why food craving acceptance exhibited one of the highest negative associations with willingness to consume a healthy diet in the model. Juarascio et al. (2011), who developed the inventory, showed that the correlation between food craving acceptance and willingness to consume a healthy diet of individuals was positive and moderate (r = 0.33; *p* < 0.001). Juarascio et al. (2011) stated that certain strategies aimed at increasing the acceptance of distressing thoughts and cognitions—and reducing avoidance strategies—theoretically correspond to lower episodes of overeating by fostering psychological flexibility. Moreover, Manchón et al. (2021) found that the correlation between food craving acceptance and willingness to consume a healthy diet of individuals was nearly zero (r = 0.07; *p* < 0.001). However, in the study by Hamurcu et al. (2023), who conducted the Turkish validity and reliability analysis of this inventory among university students, this association was found to be negative (r = −0.48; *p* < 0.001), consistent with our finding. Discrepancies between our findings and those reported by Juarascio et al. (2011) and Manchón et al. (2021) may stem from methodological variations and cultural differences, or how acceptance-based strategies are operationalized across different research contexts; however, such explanations remain speculative and require future empirical investigation. Furthermore, variations in participants’ psychological flexibility regarding eating-related experiences across the studies likely contributed to these divergences. An indirectly significant association was found between high food craving acceptance and low self-esteem, statistically associated through willingness to consume a healthy diet. A similar finding was also observed statistically associated through willingness to consume a healthy diet and social appearance anxiety. Given that this study is cross-sectional, these indirect pathways reflect statistical covariation rather than causal directions, and reciprocal effects cannot be ruled out. Moreover, numerous studies explained the relationship between food craving and eating attitudes (Abdella et al., 2019; Fahrenkamp et al., 2019; Tras & Gokcen, 2023). Tras and Gokcen (2023) found that individuals preoccupied with thoughts of eating and experiencing loss of control displayed statistical associations with concurrent patterns in their eating attitudes, both directly and indirectly.

A negative association was found between willingness to consume a healthy diet and social appearance anxiety in this study. It was determined that students displaying high motivation for healthy eating are observed to report low social appearance anxiety. Duran and Tras (2022) found positive significant relationships between social appearance anxiety and “focus on eating thoughts” and “loss of eating control”. Kaya et al. (2025) stated that individuals with a positive body image had lower levels of food addiction. Faraji and Firat (2022), Tan and Chow (2014), Janjetic et al. (2020), Murray et al. (2022), and Dicker-Oren et al. (2024) found that high stress and/or anxiety co-occurred with alterations in the eating behaviour patterns of individuals, showing links to higher levels of food consumption. Moreover, Manchón et al. (2021) found a non-significant relationship between anxiety and willingness to consume a healthy diet. However, Juarascio et al. (2011) found significant positive relationships between body dissatisfaction and motivation to eat healthily. Discrepancies in study results found in the literature may stem not only from differences between study groups but also from the fact that individuals possess varying sources of motivation. If individuals engage in healthy eating because they wish to feel energetic, improve their long-term quality of life, and respect their bodies—rather than to seek the approval of others—it is natural to observe a negative relationship (self-determination theory: Ryan & Deci, 2000). Conversely, high social appearance anxiety frequently co-occurs in individuals who adopt strict dietary regimens to achieve ideal body standards in order to gain others’ approval; in such cases, the desire for healthy eating may be accompanied by higher levels of body-related obsessions, explaining why a positive relationship might be found (restrained eating and restrictive cycle models: Herman & Polivy, 1980). Additionally, it was found that students reporting higher willingness to consume a healthy diet also exhibit higher self-esteem levels, a relationship statistically associated through social appearance anxiety. Moreover, the results indicate that higher food craving acceptance is indirectly associated with lower self-esteem through the sequential pathway of willingness to consume a healthy diet and social appearance anxiety, or through willingness to consume a healthy diet alone. However, Juarascio et al. (2011) and Manchón et al. (2021) reported that the self-esteem of individuals exhibited positive correlations with the increasing acceptance of food craving (r = 0.08; *p* < 0.001; and r = 0.24; *p* < 0.01, respectively). Differences in the study results may have arisen due to variations among individuals in the study groups regarding the levels of their awareness of healthy nutrition and their levels of food-related psychological flexibility, though such explanations remain speculative and warrant future empirical investigation.

In this study, we found that positive subjective evaluation of weight change is significantly associated with a higher willingness to consume a healthy diet among university students. Moreover, the subjective evaluation of weight change in students is indirectly positively related to their self-esteem level through social appearance anxiety. The literature indicates that university students with high self-esteem exhibit lower levels of fear of weight gain or weight phobia (Abdoli et al., 2025). However, we could not find any other studies in the literature that had previously examined this finding.

Higher weekly fast-food intake (such as toast, kebab, bakery items, hamburger, pizza, and French fries) was found to be significantly associated with a lower willingness to consume a healthy diet. In this study, while 9.3% (n = 36) of the university students stated that they did not consume fast-food, a large proportion—specifically 87.9% (n = 340)—reported consuming fast-food 1–6 times a week, and 2.8% (n = 11) reported consuming it seven or more times a week. Additionally, 76.4% (n = 296) of the students indicated that they did not live with their families. While it is plausible to consider that living away from home alongside traditional societal values regarding home-cooked family meals may co-occur with distinct dietary patterns, the cross-sectional nature of this study precludes any causal inferences regarding these living arrangements and fast-food consumption patterns. Similarly, El Ansari et al. (2012) stated that the eating habits of most students display distinct characteristics during university years. However, given the cross-sectional design, these cultural and living arrangement explanations should be viewed as possible interpretations requiring future investigation through more in-depth longitudinal or qualitative studies. Abdella et al. (2019) found that those experiencing fast-food cravings exhibited lower scores on deliberate food intake restriction treatments aimed at managing body weight. Additionally, the findings showed significant negative indirect associations between the fast-food intake level and self-esteem, statistically associated through willingness to consume a healthy diet and/or social appearance anxiety. Tang and Lee (2024) found that the fast-food intake level of young adults was positively associated with symptoms of depression and anxiety.

## 6. Limitations and Future Directions

This study has several limitations. First, the structural paths and hypothesized associations specified in the model reflect theoretical connections rather than demonstrated causal effects due to the cross-sectional and observational design of the study. Future longitudinal or experimental designs are needed to establish temporal ordering and causality among self-esteem, social appearance anxiety, food craving acceptance, and willingness to consume a healthy diet. Second, the sample was restricted to students recruited from randomly sampled faculties in a single state university, which limits the generalizability of the results. Future research should expand the scope by including samples from both state and private universities across multiple regions or provinces. Third, specific variables—namely, BMI, weekly fast-food intake level, and subjective evaluation of weight change—relied on self-reported single-item observed variables. Moreover, weekly fast-food intake was treated as an ordinal variable rather than continuous, and subjective evaluation of weight change as a binary variable—positive/negative. Another limitation is that, despite our initial probability-based sampling strategy, the possibility of non-response bias cannot be entirely ruled out, as a precise response rate could not be definitively calculated due to the survey being administered using a hybrid method (face-to-face and online). Additionally, all key psychosocial variables were self-reported and therefore subject to common-method bias and self-report measurement limitations. Although standard methodological precautions were taken during data collection, potential response bias (e.g., social desirability or underreporting regarding body shape, food cravings, and self-esteem) cannot be entirely ruled out, which may partially explain the non-significant association observed between BMI and self-esteem. An additional limitation of this study is that the internal consistency reliability values for the social appearance anxiety (CA = 0.962; CR = 0.966) slightly exceed the critical value of 0.95, suggesting a possible redundancy of items. Finally, only one item from the original scales was removed from the initial model due to low factor loading, and several potential confounders could not be controlled. Model complexity and single-item observed variables may explain the low explanatory power despite the sample-specific predictive utility observed in the PLSpredict analysis. Future studies could incorporate objective measurements and broader demographic diversity, and examine a more comprehensive range of physical, psychological, and cognitive factors influencing the psychological well-being of university students.

## 7. Conclusions

This study reveals the statistical pathways of the associations among food craving acceptance, willingness to consume a healthy diet, social appearance anxiety, BMI, subjective evaluation of weight change, fast-food intake level, and self-esteem of university students. With the exception of BMI and self-esteem, the other pathways were all found to be significant. However, contrary to our hypothesis, the structural paths between food craving acceptance and the willingness to consume a healthy diet were negative and significant. This association suggests that acceptance-based processes regarding eating behaviours may be linked to more complex or context-specific cognitive patterns rather than reflecting a direct indicator of food-related psychological flexibility. The pathway between self-esteem and social appearance anxiety had the highest value. Simultaneously, it was found that the pathways linking self-esteem–social appearance anxiety–willingness to consume a healthy diet held the greatest value compared to the other indirect pathways. Despite its high complexity and low explanatory power, the proposed conceptual model achieved medium PLS predictive power, demonstrating some out-of-sample predictive relevance within the current PLSpredict framework. However, these findings should be interpreted as predictive utility within this sample rather than broader population-level generalizability. These findings, which are exploratory in nature, establish a fundamental framework for future longitudinal research on health promotion and intervention programmes while offering valuable insights to professionals providing health and psychological guidance services to university students.

## Figures and Tables

**Table 1:** Demographic characteristics of students.

		*n*	%
Gender	Male	176	45.5
	Female	211	54.5
Place of stay	Family	91	23.5
	Friends/alone	47	12.1
	Hall of Residence	249	64.3
Income status	Income < Expense	58	15.0
	Income = Expense	213	55.0
	Income > Expense	116	30.0
Education level of mother	Primary school	238	61.5
	High school	82	21.2
	Graduate	53	13.7
	Postgraduate	14	3.6
Education level of father	Primary school	170	43.9
	High school	108	27.9
	Graduate	89	23.0
	Postgraduate	20	5.2
Status of sport participation	Yes	98	25.3
	No	289	74.7
Healthy nutrition status	Yes	148	38.2
	No	239	61.8
Alcohol use status	Yes	40	10.3
	No	347	89.7
Smoking status	Yes	85	22
	No	302	78
Nutritional preference	More meat	109	28.2
	More vegetables	89	23
	Balanced meat and vegetables	189	48.8
Weight change in recent years and how it affects people	I gained weight, and it affected me positively	46	11.9
	I lost weight, and it affected me positively	56	14.5
	There was no change in weight, and it affected me positively	78	20.2
	I gained weight, and it affected me negatively	117	30.2
	I lost weight, and it affected me negatively	47	12.1
	There was no change in weight, and it affected me negatively	43	11.1
Fast-food intake level (week)	0	36	9.3
	1–3 times	278	71.8
	4–6 times	62	16
	≥7 times	11	2.8
Probiotic/prebiotic food intake level (week)	0	7	1.8
	1–3 times	232	59.9
	4–6 times	114	29.5
	≥7 times	34	8.8
BMI (Mean ± Standard Deviation)		23.35 ± 4.63	

Fast foods: toast, kebab, various bakery items, hamburger, pizza, French fries, fried chicken pieces, chips, biscuit, etc. Probiotic/prebiotic foods: homemade yoghurt, kefir, cheese, buttermilk, pickles, vinegar, garlic, onion, banana, apple, green and yellow vegetables according to season or ready-made probiotic preparations, etc.

**Table 2:** Internal consistency reliability and convergent validity of the final PLS-SEM model.

	Cronbach’s Alpha (CA)	Composite Reliability (CR)	Average Variance Extracted (AVE)
Self-esteem	0.900	0.918	0.531
Social appearance anxiety	0.962	0.966	0.645
Food craving acceptance	0.714	0.804	0.585
Willingness to consume a healthy diet	0.811	0.864	0.518

**Table 3:** Fornell–Larcker criterion (FLc) and heterotrait–monotrait ratio (HTMT) (bootstrap 95% confidence interval) values of the final PLS-SEM model.

FLc	SAA	WCHD	BMI	SEWC	FCA	FFIL	SE
**SAA**	0.803	-	-	-	-	-	-
**WCHD**	−0.352	0.720	-	-	-	-	-
**BMI**	0.220	−0.170	1	-	-	-	-
**SEWC**	−0.303	0.222	−0.244	1	-	-	-
**FCA**	−0.051	−0.325	−0.021	0.041	0.765	-	-
**FFIL**	0.170	−0.328	0.014	−0.090	0.149	1	-
**SE**	−0.571	0.355	−0.113	0.162	−0.156	−0.151	0.729
**HTMT (Bootstrap 95%CI)**							
**WCHD**	0.399	-	-	-	-	-	-
	(0.294–0.499)						
**BMI**	0.221	0.203	-	-	-	-	-
	(0.113–0.335)	(0.127–0.313)					
**SEWC**	0.307	0.250	0.244	-	-	-	-
	(0.216–0.395)	(0.146–0.348)	(0.158–0.327)				
**FCA**	0.150 (0.105–0.272)	0.357	0.050	0.095	-	-	-
		(0.266–0.497)	(0.027–0.191)	(0.042–0.213)			
**FFIL**	0.175	0.360	0.014	0.090	0.143	-	-
	(0.072–0.278)	(0.245–0.466)	(0.002–0.123)	(0.007–0.184)	(0.062–0.264)		
**SE**	0.589 (0.497–0.675)	0.398 (0.285–0.512)	0.116 (0.061–0.231)	0.167 (0.083–0.270)	0.182 (0.135–0.292)	0.155 (0.080–0.260)	-

HTMT: heterotrait–monotrait ratio; CI: confidence interval; SE: self-esteem; SAA: social appearance anxiety; FCA: food craving acceptance; WCHD: willingness to consume a healthy diet; BMI: body mass index; SEWC: subjective evaluation of weight change; FFIL: fast-food intake level.

**Table 4:** Predictive power values of the final PLS structural model.

	PLS Model Out-of-Sample		LM Out-of-Sample		
	RMSE	MAE	RMSE	MAE	
SE1	0.598	0.482	0.587	0.455	
SE2	0.518	0.449	0.531	0.453	
SE3	0.733	0.553	0.745	0.565	
SE4	0.592	0.485	0.604	0.492	
SE5	0.734	0.576	0.731	0.568	
SE6	0.681	0.529	0.685	0.542	
SE7	0.601	0.473	0.611	0.479	
SE8	0.881	0.701	0.882	0.707	
SE9	0.847	0.681	0.869	0.721	
SE10	0.831	0.684	0.828	0.659	
	**In-Sample**		**Out-of-Sample**		
	**MSE**	**MAE**	**MSE**	**MAE**	**Overfit**
SE	0.644	0.635	0.655	0.640	0.019
SAA	0.807	0.713	0.815	0.716	0.011
WCHD	0.751	0.688	0.772	0.697	0.028

RMSE: root mean square error; MAE: mean absolute error; PLS: partial least square; LM: linear model; SE: self-esteem; SAA: social appearance anxiety; WCHD: willingness to consume a healthy diet.

**Table 5:** Direct, indirect, and total effects in the final bootstrapped PLS-SEM model.

Hypothesis/Path	Effect Type	β	Bootstrap SD	t-Value	*p*-Value	Bootstrap 95% CI	
H1: SAA→SE	Direct	−0.515	0.048	−10.757	<0.001	−0.608	−0.421
H2: WCHD→SE	Direct	0.179	0.050	3.609	<0.001	0.082	0.277
H3: BMI→SE	Direct	0.030	0.041	0.738	0.449	−0.052	0.109
H4: FCA→WCHD	Direct	−0.296	0.047	−6.300	<0.001	−0.393	−0.210
H5: WCHD→SAA	Direct	−0.285	0.047	−6.032	<0.001	−0.377	−0.193
H6: SEWC→WCHD	Direct	0.179	0.045	3.962	<0.001	0.089	0.264
H7: SEWC→SAA	Direct	−0.210	0.047	−4.432	<0.001	−0.300	−0.116
H8: FFIL→WCHD	Direct	−0.266	0.047	−5.669	<0.001	−0.355	−0.171
H9: BMI→WCHD	Direct	−0.128	0.043	−3.016	0.004	−0.211	−0.041
H10: BMI→SAA	Direct	0.120	0.052	2.321	0.022	0.016	0.221
H11a: WCHD→SAA→SE	Indirect	0.147	0.028	5.307	<0.001	0.096	0.205
H11b: FCA→WCHD→SE	Indirect	−0.053	0.017	−3.059	0.001	−0.091	−0.023
H11c: FCA→WCHD→SAA→SE	Indirect	−0.043	0.010	−4.196	<0.001	−0.067	−0.027
H11d: FCA→WCHD→SAA	Indirect	0.084	0.018	4.693	<0.001	0.054	0.124
H11e: FFIL→WCHD→SE	Indirect	−0.048	0.016	−2.973	<0.001	−0.083	−0.019
H11f: FFIL→WCHD→SAA→SE	Indirect	−0.039	0.011	−3.645	<0.001	−0.062	−0.021
H11g: FFIL→WCHD→SAA	Indirect	0.076	0.020	3.871	<0.001	0.042	0.118
H11h: SEWC→WCHD→SAA	Indirect	−0.051	0.015	−3.388	<0.001	−0.082	−0.024
H11i: SEWC→WCHD→SE	Indirect	0.032	0.012	2.588	<0.001	0.011	0.059
H11j: SEWC→WCHD→SAA→SE	Indirect	0.026	0.008	3.261	<0.001	0.012	0.043
H11k: BMI→WCHD→SAA	Indirect	0.037	0.014	2.694	0.004	0.011	0.065
H11l: BMI→SAA→SE	Indirect	−0.063	0.028	−2.205	0.022	−0.119	−0.008
H11m: BMI→WCHD→SE	Indirect	−0.023	0.010	−2.247	0.004	−0.045	−0.006
H11n: BMI→WCHD→SAA→SE	Indirect	−0.019	0.007	−2.587	0.004	−0.035	−0.006
WCHD→SE	Total	0.325	0.053	6.171	<0.001	0.221	0.429
FCA→SE	Total	−0.096	0.022	−4.471	<0.001	−0.144	−0.059
FCA→SAA	Total	0.084	0.018	4.693	<0.001	0.054	0.124
FFIL→SE	Total	−0.087	0.022	−3.970	<0.001	−0.134	−0.048
FFIL→SAA	Total	0.076	0.020	3.871	<0.001	0.042	0.118
SEWC→SAA	Total	−0.261	0.047	−5.506	<0.001	−0.352	−0.165
SEWC→SE	Total	0.166	0.029	5.719	<0.001	0.110	0.224
BMI→SAA	Total	0.157	0.055	2.836	0.005	0.046	0.264
BMI→SE	Total	−0.073	0.056	−1.303	0.184	−0.184	0.040

H1–H10: hypotheses for direct effects; H11a–H11n: hypotheses for indirect effects; β: standardised path coefficient; SD: standard deviation; CI: confidence interval; SE: self-esteem; SAA: social appearance anxiety; FCA: food craving acceptance; WCHD: willingness to consume a healthy diet; BMI: body mass index; SEWC: subjective evaluation of weight change; FFIL: fast-food intake level.

## Data Availability

The data that support the findings of this study are available from the corresponding author upon reasonable request.

## Supplementary Materials

The following supporting information can be downloaded at: https://www.mdpi.com/article/10.3390/bs16101959/s1, Table S1: Internal consistency reliability and convergent validity of the initial PLS-SEM model; Table S2: Fornell–Larcker criterion (FLc) and heterotrait–monotrait ratio (HTMT) (bootstrap 95% confidence interval) values of the initial PLS-SEM model; Table S3: Direct, indirect, and total effects in the initial bootstrapped PLS-SEM model; Figure S1: The initial PLS structural equation model. λ: factor loading; β: standardised path coefficient; straight line arrow: positive relationship; straight dashed line arrow: negative relationship; SE: self-esteem; SAA: social appearance anxiety; FCA: food craving acceptance; WCHD: willingness to consume a healthy diet; BMI: body mass index; SEWC: subjective evaluation of weight change; FFIL: fast-food intake level.

## Abbreviations

PLS-SEM	Partial least squares structural equation modelling
CB-SEM	Covariance based SEM
BMI	Body mass index
H	Hypothesis
SE	Self-esteem
FCA	Food craving acceptance
WCHD	Willingness to consume a healthy diet
SAA	Social appearance anxiety
SEWC	Subjective evaluation of weight change
FFIL	Fast-food intake level (weekly)
VIF	Variance inflation factor
AVE	Average variance extracted
CA	Cronbach’ alpha
CR	Composite reliability
FLc	Fornell–Larcker criterion
HTMT	Heterotrait–monotrait ratio
β	Standardised path coefficient
R^2^	Determination coefficient
f2	Effect size
RMSE	Root mean square error
MAE	Mean absolute error
LM	Linear model
SRMR	Standardised root mean squared residual
