# Supplementary material for: Investigation into Effective Factors Affecting the Self-Esteem of University Students Using a PLS-SEM Model
Source: Behav Sci (Basel). 2026 Oct 21;16(10):1959. doi: 10.3390/bs16101959 (PMC13701546; doi:10.3390/bs16101959)
Supplement: Supplementary file 1 [file behavsci-16-01959-s001.zip › behavsci-4542526-supplementary.pdf]

Supplementary Materials

Investigation into Effective Factors Affecting the Self-Esteem of University Students using A PLS-SEM Model
Manuscript ID: behavsci-4542526

Content: The initial bootstrapped PLS-SEM model results (Table S1-S3, Figure S1)

Table S1. Internal consistency reliability and convergent validity of the initial PLS-SEM model.

	Cronbach's Alpha (CA)	Composite Reliability (CR)	Average Variance Extracted (AVE)
Self-esteem	0.900	0.918	0.531
Social appearance anxiety	0.962	0.966	0.645
Food craving acceptance	0.715	0.733	0.443
Willingness to consume a healthy diet	0.811	0.864	0.518

Table S2. Fornell–Larcker criterion (FLc) and heterotrait–monotrait ratio (HTMT) (bootstrap 95% confidence interval) values of the initial PLS-SEM model.

**Fornell-Larcker**	**SAA**	**WCHD**	**BMI**	**SEWC**	**FCA**	**FFIL**	**SE**
**SAA**	0.803	-	-	-	-	-	-
**WCHD**	-0.352	0.720	-	-	-	-	-
**BMI**	0.220	-0.170	1	-	-	-	-
**SEWC**	-0.303	0.222	-0.244	1	-	-	-
**FCA**	-0.025	-0.345	-0.009	0.020	0.666	-	-
**FFIL**	0.170	-0.328	0.014	-0.090	0.164	1	-
**SE**	-0.571	0.355	-0.113	0.162	-0.169	-0.151	0.729
**HTMT (Bootstrap 95%CI)**							
**WCHD**	0.399<br>(0.294-0.509)	-	-	-	-	-	-
**BMI**	0.221<br>(0.119-0.332)	0.203<br>(0.125-0.314)	-	-	-	-	-
**SEWC**	0.307<br>(0.218-0.393)	0.250<br>(0.143-0.346)	0.244<br>(0.159-0.318)	-	-	-	-
**FCA**	0.214<br>(0.156-0.332)	0.354<br>(0.286-0.481)	0.082<br>(0.048-0.222)	0.149<br>(0.085-0.263)	-	-	-
**FFIL**	0.175<br>(0.069-0.282)	0.360<br>(0.239-0.474)	0.014<br>(0.002-0.118)	0.090<br>(0.007-0.180)	0.145<br>(0.082-0.246)	-	-
**SE**	0.589<br>(0.501-0.674)	0.398<br>(0.283-0.520)	0.116<br>(0.062-0.227)	0.167<br>(0.085-0.267)	0.187<br>(0.155-0.284)	0.155<br>(0.078-0.270)	-

HTMT: heterotrait–monotrait ratio; CI: confidence interval; SE: self-esteem; SAA: social appearance anxiety; FCA: food craving acceptance; WCHD: willingness to consume a healthy diet; BMI: body mass index; SEWC: subjective evaluation of weight change; FFIL: fast-food intake level.

Table S3. Direct, indirect, and total effects in the initial bootstrapped PLS-SEM model.

Hypothesis/Path	Effect type	β	Bootstrap SD	t-value	p-value	Bootstrap 95% CI	
H1: SAA→SE	Direct	-0.515	0.048	-10.764	<0.001	-0.608	-0.421
H2: WCHD→SE	Direct	0.178	0.050	3.597	<0.001	0.081	0.277
H3: BMI→SE	Direct	0.030	0.041	0.738	0.449	-0.052	0.109
H4: FCA→WCHD	Direct	-0.307	0.059	-5.200	0.008	-0.401	-0.231
H5: WCHD→SAA	Direct	-0.285	0.047	-6.031	<0.001	-0.377	-0.192
H6: SEWC→WCHD	Direct	0.174	0.045	3.891	<0.001	0.084	0.257
H7: SEWC→SAA	Direct	-0.210	0.047	-4.431	<0.001	-0.300	-0.116
H8: FFIL→WCHD	Direct	-0.260	0.047	-5.501	<0.001	-0.348	-0.163
H9: BMI→WCHD	Direct	-0.126	0.042	-2.978	0.004	-0.208	-0.038
H10: BMI→SAA	Direct	0.120	0.052	2.319	0.022	0.016	0.221
H11a: WCHD→SAA→SE	Indirect	0.147	0.028	5.307	<0.001	0.096	0.205
H11b: FCA→WCHD→SE	Indirect	-0.055	0.019	-2.836	0.009	-0.094	-0.023
H11c: FCA→WCHD→SAA→SE	Indirect	-0.045	0.012	-3.689	0.008	-0.070	-0.028
H11d: FCA→WCHD→SAA	Indirect	0.088	0.022	3.946	0.008	0.056	0.130
H11e: FFIL→WCHD→SE	Indirect	-0.046	0.016	-2.967	<0.001	-0.081	-0.019
H11f: FFIL→WCHD→SAA→SE	Indirect	-0.038	0.011	-3.624	<0.001	-0.061	-0.020
H11g: FFIL→WCHD→SAA	Indirect	0.074	0.019	3.861	<0.001	0.040	0.115
H11h: SEWC→WCHD→SAA	Indirect	-0.050	0.015	-3.391	<0.001	-0.080	-0.023
H11i: SEWC→WCHD→SE	Indirect	0.031	0.012	2.585	<0.001	0.011	0.057
H11j: SEWC→WCHD→SAA→SE	Indirect	0.026	0.008	3.254	<0.001	0.011	0.042
H11k: BMI→WCHD→SAA	Indirect	0.036	0.013	2.688	0.004	0.011	0.063
H11l: BMI→SAA→SE	Indirect	-0.062	0.028	-2.204	0.022	-0.119	-0.008
H11m: BMI→WCHD→SE	Indirect	-0.023	0.010	-2.240	0.005	-0.045	-0.005
H11n: BMI→WCHD→SAA→SE	Indirect	-0.019	0.007	-2.576	0.004	-0.034	-0.005
WCHD→SE	Total	0.325	0.053	6.163	<0.001	0.221	0.429
FCA→SE	Total	-0.100	0.026	-3.886	0.008	-0.150	-0.062
FCA→SAA	Total	0.088	0.022	3.946	0.008	0.056	0.130
FFIL→SE	Total	-0.085	0.021	-3.945	<0.001	-0.131	-0.046
FFIL→SAA	Total	0.074	0.019	3.861	<0.001	0.040	0.115
SEWC→SAA	Total	-0.260	0.047	-5.475	<0.001	-0.350	-0.163
SEWC→SE	Total	0.165	0.029	5.672	<0.001	0.108	0.222
BMI →SAA	Total	0.156	0.055	2.825	0.005	0.046	0.264
BMI →SE	Total	-0.073	0.056	-1.291	0.190	-0.183	0.040

H1-H10: hypotheses for direct effects; H11a-H11n: hypotheses for indirect effects; β: standardised path coefficient; SD: standard deviation; CI: confidence interval; SE: self-esteem; SAA: social appearance anxiety; FCA: food craving acceptance; WCHD: willingness to consume a healthy diet; BMI: body mass index; SEWC: subjective evaluation of weight change; FFIL: fast-food intake level.

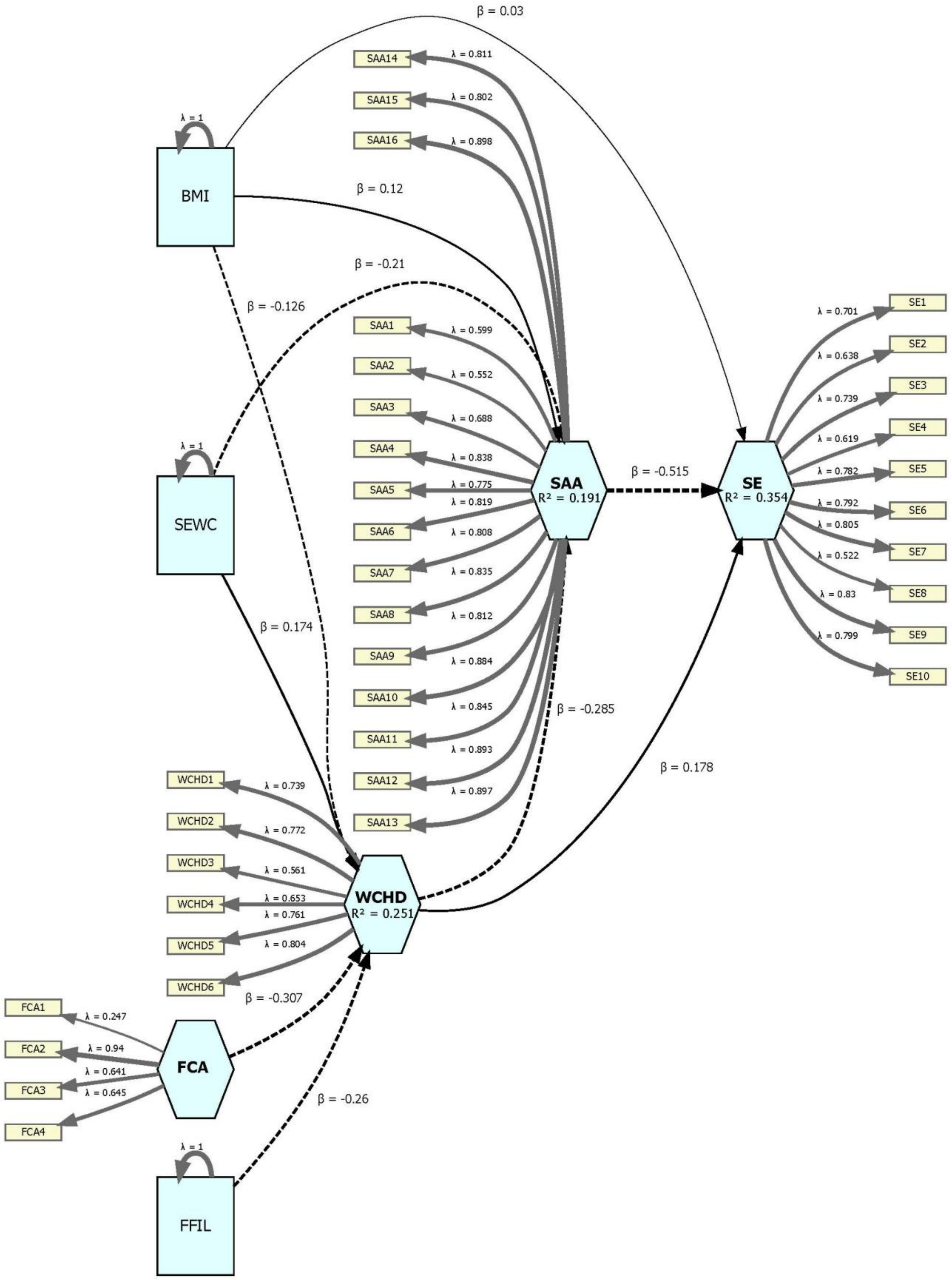

Figure S1. The initial PLS structural equation model. λ: factor loading; β: standardised path coefficient; straight line arrow: positive relationship; straight dashed line arrow: negative relationship; SE: self-esteem; SAA: social appearance anxiety; FCA: food craving acceptance; WCHD: willingness to consume a healthy diet; BMI: body mass index; SEWC: subjective evaluation of weight change; FFIL: fast-food intake level.
